# Transcriptomic atlas for hypoxia and following re-oxygenation in *Ancherythroculter nigrocauda* heart and brain tissues: insights into gene expression, alternative splicing, and signaling pathways

**DOI:** 10.3389/fgene.2024.1365285

**Published:** 2024-04-16

**Authors:** Jian Chen, Zhen Hu, Pei Li, Guiying Wang, Huijie Wei, Qing Li, Beide Fu, Yanhong Sun

**Affiliations:** ^1^ Fisheries Research Institute, Wuhan Academy of Agricultural Sciences, Wuhan, China; ^2^ Hubei Provincial Fisheries Technology Extension Center, Wuhan, China; ^3^ Ruibiao (Wuhan) Biotechnology Co., Ltd, Wuhan, China

**Keywords:** hypoxia, transcriptome, recovery, multi-tissue, Ancherythroculter nigrocauda

## Abstract

Hypoxia is a mounting problem that affects the world’s freshwaters, with severe consequence for many species, including death and large economical loss. The hypoxia problem has increased recently due to the combined effects of water eutrophication and global warming. In this study, we investigated the transcriptome atlas for the bony fish *Ancherythroculter nigrocauda* under hypoxia for 1.5, 3, and 4.5 h and its recovery to normal oxygen levels in heart and brain tissues. We sequenced 21 samples for brain and heart tissues (a total of 42 samples) plus three control samples and obtained an average of 32.40 million raw reads per sample, and 95.24% mapping rate of the filtered clean reads. This robust transcriptome dataset facilitated the discovery of 52,428 new transcripts and 6,609 novel genes. In the heart tissue, the KEGG enrichment analysis showed that genes linked to the Vascular smooth muscle contraction and MAPK and VEGF signaling pathways were notably altered under hypoxia. Re-oxygenation introduced changes in genes associated with abiotic stimulus response and stress regulation. In the heart tissue, weighted gene co-expression network analysis pinpointed a module enriched in insulin receptor pathways that was correlated with hypoxia. Conversely, in the brain tissue, the response to hypoxia was characterized by alterations in the PPAR signaling pathway, and re-oxygenation influenced the mTOR and FoxO signaling pathways. Alternative splicing analysis identified an average of 27,226 and 28,290 events in the heart and brain tissues, respectively, with differential events between control and hypoxia-stressed groups. This study offers a holistic view of transcriptomic adaptations in *A. nigrocauda* heart and brain tissues under oxygen stress and emphasizes the role of gene expression and alternative splicing in the response mechanisms.

## 1 Introduction

Oxygen supply is a vital factor for animals because oxygen is used to produce energy through the cellular respiration process. Lack of oxygen (hypoxia) quickly leads to ATP depletion and seriously damages various organs in a short time. In aquaculture, hypoxia is a main environmental threat to freshwater and marine animals. Hypoxia not only limits the biodiversity in a specific area (Vaquer-Sunyer and Duarte, 2008), but also restricts the growth of many species (Bianchi et al., 2010; Hou et al., 2020). An in-depth understanding of hypoxia tolerance in fishes, and how it varies among individuals and species, is crucial for accurately predicting the impacts of hypoxia on fish populations and production (Rogers et al., 2016).

During the long evolution history, natural selection has allowed fishes to develop a range of adaptations to variable oxygen concentrations (Xiao, 2015). Fishes that depend on aerobic metabolism for rapid swimming, such as sailfish, tuna, and salmon, are moderate to extremely sensitive to anoxia (*Istiophorus platypterus*) (Svendsen et al., 2016), tuna (*Thunnus maccoyii*) (Fitzgibbon et al., 2010) and salmon (*Oncorhynchus kisutch*) (Clark et al., 2013), are moderate to extreme sensitive to anoxia. Whereas some carps, eels and hagfish are well adapted to hypoxia conditions. Even closely related fish species that inhabit the same environment exhibit varied adaption to oxygen concentrations (Burggren et al., 2019; Fu et al., 2021; Wang et al., 2022). In previous studies, body mass was found to play a role in fish hypoxia tolerance (Verberk et al., 2022). Larger fish seem to be more hypoxia-tolerant than smaller fish in short-term laboratory settings, and often show enhanced swimming capacity in hypoxic conditions (Müller et al., 2023).

Transcriptomic studies have been conducted about hypoxic stress in many fish species. In blunt snout bream (*Megalobrama amblycephala*) liver tissue, KEGG pathway enrichment analysis found that these differentially expressed genes (DEGs) were enriched in HIF-1, FOXO, MAPK, PI3K-Akt and AMPK signaling pathway (Zhao et al., 2022). While in another Cyprinidae fish, silver carp (*Hypophthalmichthys molitrix*), KEGG enrichment analysis found a similar pattern that PI3K-Akt, FOXO and JAK-STAT were among most prominent pathway activated by hypoxia in heart tissue (Li et al., 2021). In Nile tilapia (*Oreochromis niloticus*) heart tissue, 289 differential expressed genes (DEGs) and 103 genes with differential usage of exons and splice junction events were identified (Xia et al., 2018). In large yellow croaker (*Larimichthys crocea*) gill and heart tissues, a total of 1,546 and 2,746 DEGs were found 6, 24, and 48 h after the fish were exposed to hypoxia stress. Among these DEGs, innate immunity-related genes were found to be downregulated, whereas DEGs in the glycolysis pathway and tricarboxylic acid cycle were up-regulated (Mu et al., 2020). DEGs were identified in brain tissue of *Takifugu rubripes* (Shang et al., 2022). In spotted sea bass (*Lateolabrax maculatus*) liver tissue, DEGs enriched in hypoxia-inducible factor (HIF-1), apoptosis, and purine metabolism were found after the fish were exposed to hypoxia for 12 h (Yan et al., 2021). In largemouth bass (*Micropterus salmoides*) liver tissue, DEGs related to glucose and lipid metabolism were enriched in fish exposed to hypoxic stress for 24 h then to reoxygenation for 12 h (Sun et al., 2020).

*Ancherythroculter nigrocauda* is a promising aquaculture species in Asia. It has a large body size and good hypoxia tolerance compared with other Cyprinidae species, such as barbel chub (*Squaliobarbus curriculus*) and silver carp (*Hypophthalmichthys molitrix*) (Jian et al., 2020). But with high density rearing, which is becoming increasingly popular in fish farming, undesirable hypoxia caused by water bloom, high temperatures, and other reasons can cause huge economic loss for cultivated fish. Therefore, breeding an *A. nigrocauda* strain with high hypoxia tolerance is critical. For this, a deeper understanding of the transcriptomic atlas related to hypoxia stress for different tissues is urgently required. In this study, we investigated gene expression, alternative splicing, and signaling pathways related to acute hypoxia and following re-oxygenation in heart and brain tissues of *A. nigrocauda*. The results will provide important candidate markers for molecular-assisted breeding of this important aquaculture species.

## 2 Materials and methods

### 2.1 Sample preparation

This study was approved by the Animal Care and Use Committee of the Wuhan Agriculture Academy.

The fish used in this study (body length 38.5 ± 2.46 cm; weight 502 ± 55.7 g; 2 years post-hatch) were obtained from the Fisheries Research Institute, Wuhan Academy of Agricultural Sciences. The fish were maintained in indoor fiberglass tanks (volume 500 L; water depth 80 cm) with a water circulating system for 30 days before use. The water was maintained at 25°C–28°C and dissolved oxygen (DO) was approximately 6 mg O_2_/L. DO was measured using a DO meter (YSI, Canada), and the photoperiod in the rearing room was 12-h dark/12-h light.

To begin the hypoxia experiment, 30 fish were transferred to a single 100-L tank for temporary rearing with an air pump. After 8 h, the DO in the water was 6 mg O_2_/L and three fish was collected as the normal control group (0 h). Then, the DO was lowered to 0.5 mg/L over 15 min. The brain and heart tissues of three randomly selected fish were collected at 1.5, 3, and 4.5 h after the hypoxia challenge as the hypoxia groups ((1.5h, 3h, 4.5 h). At each time point, three other fish were transferred to another tank with DO 4.0 mg/L for 2 h of re-oxygenation as the re-oxygenation groups (Re1.5h, Re3h, Re4.5 h). All the collected tissue samples were put in liquid nitrogen for 10 min, then transferred to RNase-free tubes for preservation.

### 2.2 Library construction and sequencing

Total RNA was extracted from each tissue sample using TRIzol reagent (Invitrogen, UK) following the manufacturer’s protocol. The quality of the extracted RNA was assessed by Nanodrop-2000 (Thermo Scientific, United States) and BioAnalyzer 2,100 (Agilent). Samples with RNA integrity numbers >6 were kept for RNA-Seq library construction using a MGIEasy RNA Library Prep Kit (BGI, China) following the manufacturer’s instructions. The qualified libraries were sequenced using a DNBSEQ platform in paired-end 150 mode to obtain approximately 60 million paired-end reads per sample. The raw data for all 42 libraries have been deposited in the NCBI SRA database under accession number PRJNA1027844.

### 2.3 Sequence read processing and annotation update

Raw sequencing data were quality checked using FastQC v0.11.9 (Davis et al., 2021). Then, fastp v0.21.0 was used with default parameters to filter low-quality reads. The filtered reads were checked again with FastQC. After removing low quality bases and reads, the filtered reads were mapped to the *A. nigrocauda* reference genome (Sun et al., 2021) using STAR v2.7.9a (Dobin et al., 2013) with default parameters. Reference-based transcript assembly was carried out for each sample using StringTie v2.1.4 (Pertea et al., 2015). And the result gff file for each sample were merged by StringTie merge to generate an updated annotation file for the genome. Finally, gffcompare v0.12.1 (Pertea and Pertea, 2020) was used to compared the differences between the updated annotation files and previous one to identify novel transcripts and genes.

### 2.4 Identification and enrichment analysis of the DEGs

After the annotation update, the following analyses were carried out in R v4.0 (https://www.R-project.org/). Read count for each gene in different libraries was obtained using the Rsubread v2.12.3 package (Liao et al., 2019). Gene counts were filtered for lowly expressed genes with at least three samples being required to have >5 reads. Then, nine comparison were carried out for the brain and heart tissues using DESeq2 v1.26.0 (Love et al., 2014). Only genes with |log2 (fold_change)| >1 and FDR (false discovery rate) ≤0.05 were considered to be DEGs. Gene ontology (GO) enrichment analysis was performed using the TopGO v2.52.0 package, and Kyoto Encyclopedia of Genes and Genomes (KEGG) enrichment analysis was performed using the KEGGREST v1.40.0 package. Enriched GO and KEGG terms and pathways were identified using a hypergeometric distribution test. A *p*-value of 0.05 was used as the threshold value to determine significance.

### 2.5 Weighted correlation network analysis (WGCNA)

A weighted gene co-expression network was constructed using the WGCNA package (Langfelder and Horvath, 2008) in R v4.0. After filtering lowly expressed genes, expression data for the remaining genes in each tissue were imported into WGCNA to detect co-expression modules, which are gene clusters with similar expression pattern among all the libraries. Genes are hierarchically clustered by the topological overlap-based dissimilarity measure (Zhang and Horvath, 2005) and then a gene dendrogram is constructed based on the topological overlap matrix. To identify biologically significant modules, all the module eigengenes were used to calculate the correlation coefficients. GO and KEGG enrichment analysis was carried out for the genes in each module using the same software that was used for the DEG analysis.

### 2.6 Identification of alternative splicing (AS) in heart and brain tissues

Using the GTF files generated from the BAM mapping results, the identified AS events were classified into five groups, namely, skipped exon, alternative 5′splice site, alternative 3′splice site, mutually exclusive exons, and retained intron using rMATS v4.1.1 (Shen et al., 2014). Only AS events that were present in at least two samples of one tissue were considered stable AS events for differential AS analysis in rMATS v4.1.1 (Shen et al., 2014).

## 3 Results

### 3.1 RNA-Seq data for brain and heart tissues

In this study, we sequenced heart and brain tissue samples from three hypoxia groups (1.5h, 3h, 4.5 h), three re-oxygenation groups (Re1.5h, Re3h, Re4.5 h), and three samples as the control group (0 h). Therefore, 21 samples were sequenced for each tissue; a total of 42 samples overall. An overview of the whole experiment is provided in Supplementary Table S1. An average of 32.40 million raw reads was acquired for each sample. The Q20 for the filtered reads was 97.83%. The GC content in each library was approximately 43.03% and normally distributed. Approximately 95.24% of the filtered reads mapped to the *A. nigrocauda* reference genome (Sun et al., 2021) (Supplementary Table S1).

We used the high-quality mapped reads to update the gene annotation file for *A. nigrocauda*. The updated annotation included 52,428 new transcripts; among them, 2,694 transcripts were within the introns of previously annotated transcripts, 36,187 shared at least one exon with previously annotated transcripts, 953 overlapped with an exon on the same strand, 9,411 had been annotated previously, and 3,183 overlapped with an exon on the other strand (Supplementary Figure S1). We also annotated 6,609 new genes (Supplementary Figure S2).

### 3.2 Differentially expressed genes (DEGs) in heart tissue

**FIGURE 1:**
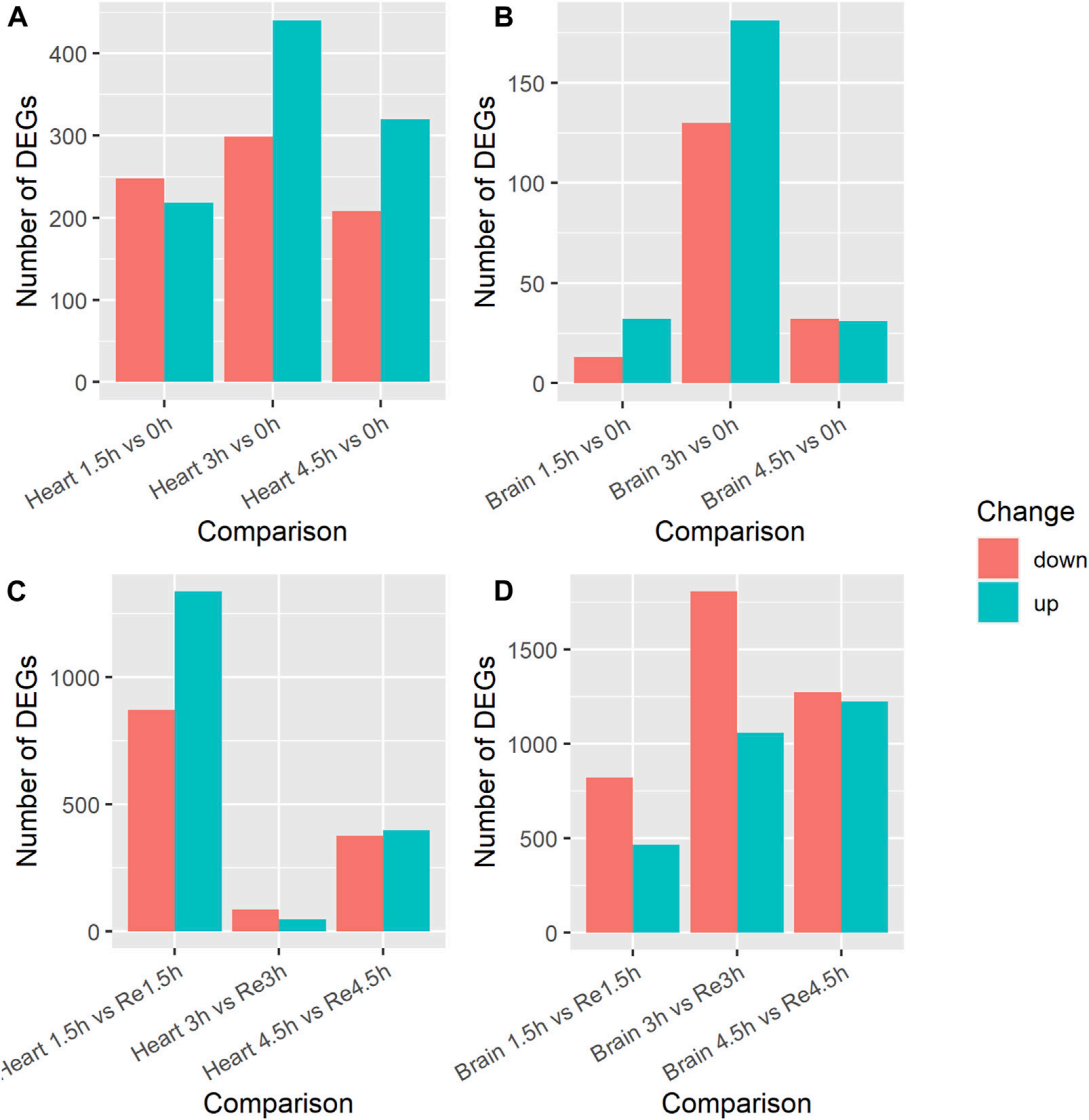
Number of differentially expressed genes (DEGs) in brain and heart tissue under acute hypoxia stress and re-oxygenation in Ancherythroculter nigrocauda. **(A)** DEGs between different time of hypoxia and control group in heart tissue. **(B)** DEGs between different time of hypoxia and control group in brain tissue. **(C)** DEGs between different time of hypoxia and 2 h re-oxygenation in heart tissue. **(D)** DEGs between different time of hypoxia and 2 h re-oxygenation in brain tissue.

We identified DEGs in the heart tissues by comparing the transcripts in the control group (0 h) with those in the three hypoxia groups (1.5h, 3h, 4.5 h), a total of three comparisons, to study the effects of exposure time on hypoxia stress. In the 1.5 h vs. 0 h comparison, 466 DEGs were identified (Supplementary Table S2); 218 were upregulated and 248 downregulated in the 1.5 h group (Figure 1A). The KEGG enrichment analysis showed that seven of these DEGs (*MYLK4*, *mylk3*, *PRKG1*, *nppa*, *avpr1aa*, *PPP1CA*) were enriched in the Vascular smooth muscle contraction pathway (ko04270). In the 3 h vs. 0 h comparison, 739 DEGs were identified (Supplementary Table S3); 440 were upregulated and 299 were downregulated in the 3 h group. Four of these DEGs (*mylk3*, *MYLK4*, *PRKG1*, *avpr1aa*) were enriched in the Vascular smooth muscle contraction (ko04270) pathway. In the 4.5 h vs. 0 h comparison, 528 DEGs were identified (Supplementary Table S4); 320 were upregulated and 208 were downregulated in the 4.5 h group. Fifteen of these DEGs (*igf1ra*, *mknk2b*, *abhd6b*, *arrb1*, *gadd45bb*, *sos2*, *MECOM*, *angpt1*, *tgfbr1b*, *mknk2a*, *gadd45ab*, *gadd45ba*, *pdgfrb*) were enriched in the MAPK signaling pathway (ko04010) and four (*pxna*, *zgc:101699*, *casp9*, *shc2*) were enriched in the VEGF signaling pathway (ko04370). Among the three comparisons, 37 DEGs were co-up-regulated, including the hypoxia inducible factor gene *hif1al*, and 18 were downregulated in all three comparisons.

We also compared the transcripts in the three hypoxia groups (1.5h, 3h, 4.5 h) with those in the re-oxygenation groups (Re1.5h, Re3h, Re4.5 h) to study the effects of re-oxygenation after different lengths of exposure to hypoxia stress. In the 1.5 h vs. Re1.5 h comparison, 2,210 DEGs were identified (Supplementary Table S5); 1,338 were upregulated and 872 were downregulated in the Re1.5 h group (Figure 1C). The GO enrichment analysis showed that nine of these DEGs (*baal1b*, *dnaja1*, *rrh*, *coch*, *myo6b*, *vegfaa*, *cpeb4a*, *vegfd*) were enriched in response to abiotic stimulus (GO:0009628) and 10 (*socs1a*, *rnft2*, *usp1*, *relt*, *enpp4*, *enpp5*, *sesn1*) were enriched in regulation of response to stress (GO:0080134). In the 3 h vs. Re3h comparison, 131 DEGs were identified (Supplementary Table S6); 47 were upregulated and 84 were downregulated in the Re3h group. In the 4.5 h vs. Re4.5 h comparison, 772 DEGs were identified (Supplementary Table S7); 397 were upregulated and 375 were downregulated in the Re4.5 h group. The KEGG enrichment analysis showed that 13 of them (*pik3r1*, *gadd45bb*, *ntrk1*, *kdsr*, *nfkbiab*, *mcl1b*, *PIK3CA*, *mcl1a*, *atf4b*, *lmna*, *casp9*, *ctsl.1*) were enriched in the Apoptosis pathway (ko:04210) and eight (*ddit4*, *pik3r1*, *fzd2*, *fzd7a*, *fzd8a*, *PIK3CA*, *wnt5b*, *PRKAA2*) were enriched in the mTOR signaling pathway (ko04150). Nine DEGs were downregulated and two were upregulated in all three comparisons.

### 3.3 Differentially expressed genes (DEGs) in brain tissue

Brain tissue is one of the most oxygen-sensitive organs in fish. We performed the same three comparisons between the control group (0 h) and the three hypoxia groups (1.5h, 3h, 4.5 h). In the 1.5 h vs. 0 h comparison, 45 DEGs were identified (Supplementary Table S8); 32 were upregulated and 13 were downregulated in the 1.5 h group (Figure 1B). In the 3 h vs. 0 h comparison, 311 DEGs were identified (Supplementary Table S9); 181 were upregulated and 130 were downregulated in the 3 h group. The KEGG enrichment analysis showed that five of these genes (*acsl4b*, *SGSM1*, *ppardb*, *angptl4*) were enriched in the PPAR signaling pathway (ko03320). In the 4.5 h vs. 0 h comparison, 63 DEGs were identified (Supplementary Table S10); 31 were upregulated and 32 were downregulated in the 4.5 group. Two of these DEGs (*pxna*, *zgc:101699*) were enriched in the VEGF signaling pathway and two (*stmn1a*, *zgc:101699*) were enriched in the MAPK signaling pathway. Only six DEGs were upregulated in all three comparisons, but none of them overlapped with the 37 DEGs that were commonly upregulated in the heart tissue comparisons.

We also compared the brain transcripts in the three hypoxia groups (1.5h, 3h, 4.5 h) with those in the re-oxygenation groups (Re1.5h, Re3h, Re4.5 h). In the 1.5 h vs. Re1.5 h comparison, 1,282 DEGs were identified (Supplementary Table S11); 462 were upregulated and 820 were downregulated (Figure 1D). The KEGG enrichment analysis showed that some of these DEGs were enriched in the mTOR signaling pathway (ko04150), Fructose and mannose metabolism (ko00051), and Wnt signaling pathway (ko04310). In the 3 h vs. Re3h comparison, 2,865 DEGs were identified (Supplementary Table S12); 1,058 were upregulated and 1,807 were downregulated. Some of these DEGs were enriched in Cytokine-cytokine receptor interaction (ko04060), the PPAR signaling pathway (ko03320), FoxO signaling pathway (ko04068), and Lysosome pathway (ko04142). In the 4.5 h vs. Re4.5 h comparison, 2,394 DEGs were identified (Supplementary Table S13); 1,224 were upregulated and 1,270 were downregulated. Eighteen DEGs were downregulated in all three comparisons, whereas none of the DEGS were commonly upregulated. Among the 18 downregulated DEGs, only one (*angptl4*, angiopoietin-related protein 4) was among the nine DEGs commonly downregulated in heart tissue.

### 3.4 Weighted gene co-expression network analysis (WGCNA) in heart tissue

**FIGURE 2:**
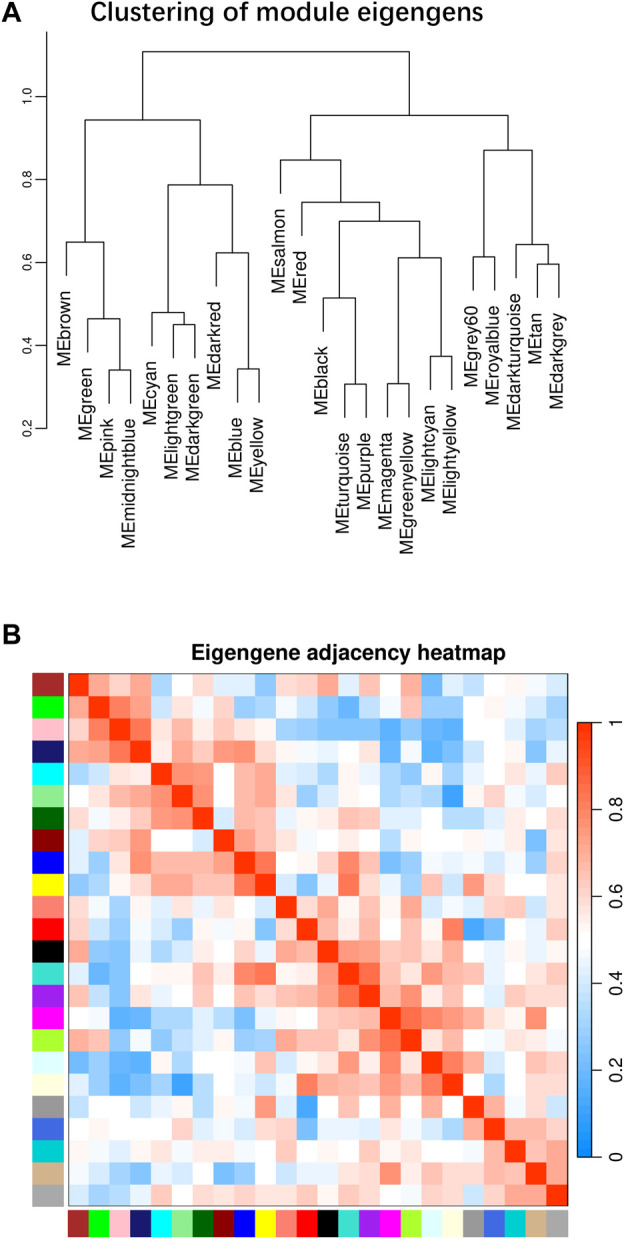
WGCNA plot for all 21 libraries in heart tissues. **(A)** Clustering of module eigengens based on gene expression value. **(B)** Eigengen adjacency heatmap for all modules.

**FIGURE 3:**
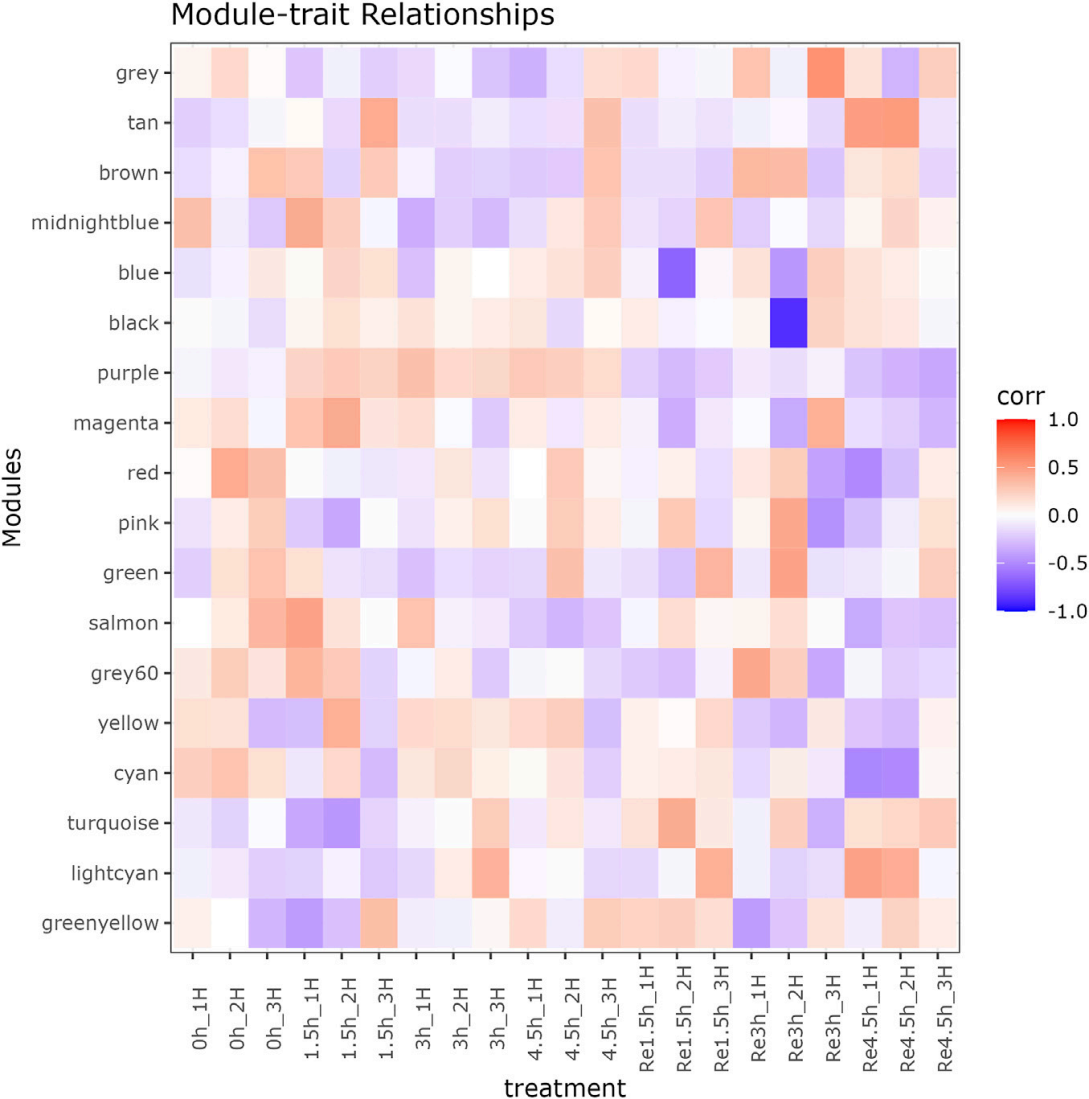
Module-trait relationship between 18 modules and 21 RNA-seq libraries in heart tissue.

Beside the single genes associated with hypoxia-resistance, we also constructed a gene network for heart tissue by WGCNA. Only genes that were expressed in at least 11 of the samples (half of all the heart tissue samples) were used for normalization. No significant differences were detected among all the libraries (Supplementary Figure S3). A gene dendrogram was generated based on the gene expression data from all 21 libraries, with each branch being defined as a module (Figure 2A). Module eigen genes in each module were used to represent its expression level. Fifteen distinct modules were identified, and their sizes ranged from 43 to 252 genes (Supplementary Table S14). A heatmap was generated to show the correlations between different modules and groups (Figure 2B). We found that the purple module was positively correlated with all three hypoxia-stressed groups (1.5h, 3h, 4.5 h) and negatively correlated with the control group and all three re-oxygenation groups (Re1.5h, Re3h, Re4.5 h) (Figure 3). The genes in the purple module were enriched in insulin receptor binding (GO:0005158) and insulin receptor signaling pathway (GO:0008286) (Supplementary Figure S4). The salmon module was negatively correlated with 4.5 h and Re4.5 h (*p* < 0.05). The genes in the salmon module were enriched in immune system process (GO:0002376), regulation of heart rate (GO:0002027), and immunoglobulin receptor binding (GO:0034987) (Supplementary Figure S5). The normalized expression for all genes in these two modules are shown in Supplementary Figure S6.

### 3.5 Alternative splicing (AS) in heart and brain tissues

**FIGURE 4:**
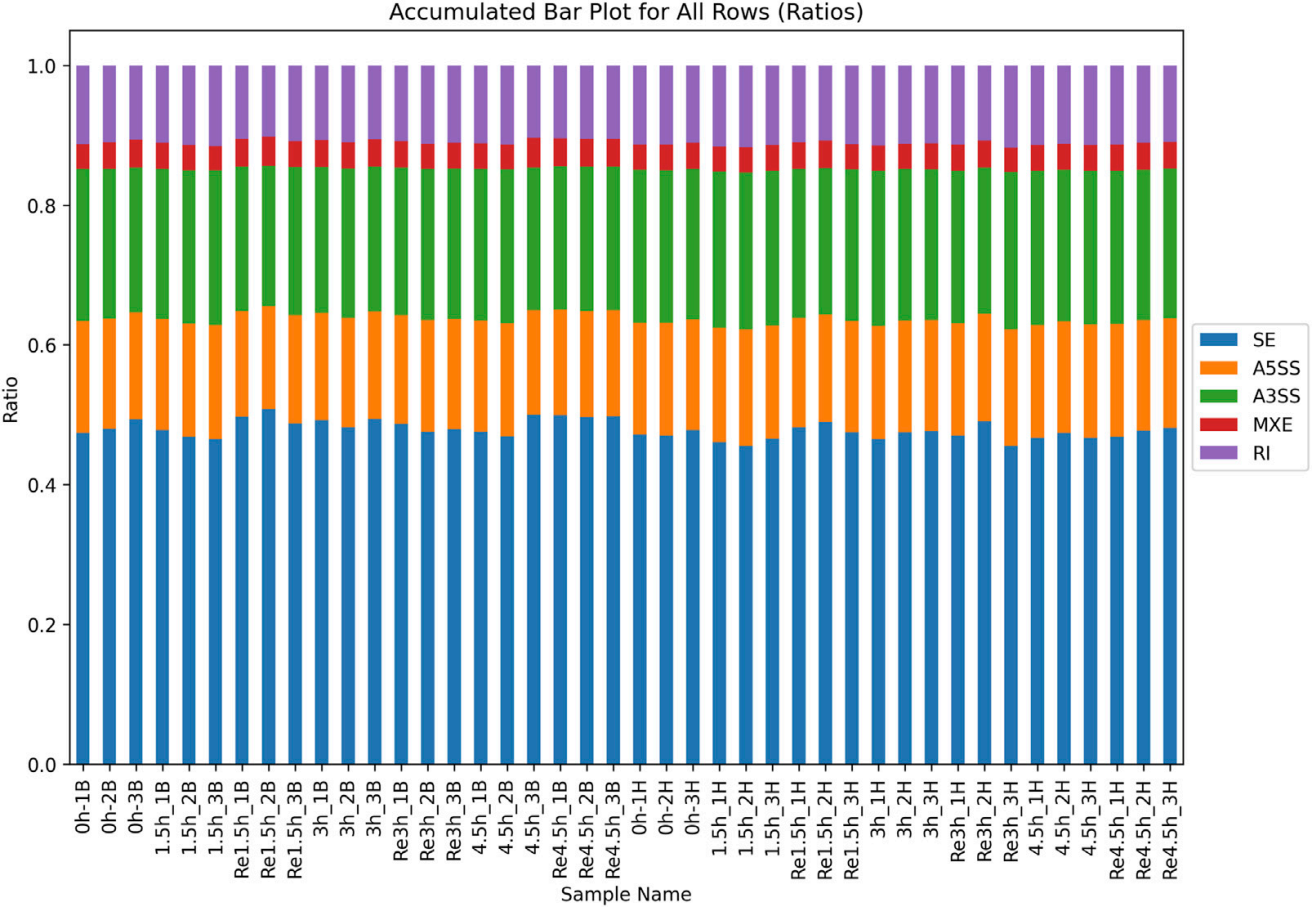
Distribution of all 5 types of AS in all samples. Skipped exon (SE) was the most common event in all samples.

For the non-DEGs, different transcripts obtained by AS may be selected according to various situations. There were an average of 27,226 and 28,290 AS events in heart and brain tissues, respectively (Supplementary Table S15). All AS events were classified into five types according to the different splicing patterns. Skipped exon was the most common event, and mutually exclusive exon was the least common event (Figure 4). All the AS events were associated with 9,902 and 9,817 genes in heart and brain tissue, respectively. To investigate the influence of hypoxia stress on AS, we compared the control group with the three hypoxia-stressed groups in both heart and brain tissues. In heart tissue, we detected 13, 22, and seven differential AS events between the control group and the 1.5h, 3h, and 4.5 h groups, respectively. In brain tissue, we detected 21, 24, and 22 differential AS events between the control group and the 1.5h, 3h, and 4.5 h groups, respectively (Supplementary Table S16).

## 4 Discussion

Acute hypoxia in aquaculture fishes can be extremely detrimental to their survival and wellbeing, leading to stress, impaired growth, and even mass mortalities if not promptly addressed (Ton et al., 2003). Acute hypoxia can be triggered by various factors including elevated water temperatures, excessive organic matter in the water, or overcrowding, which increases oxygen consumption and depletes available oxygen levels. Adjusting their metabolic pattern is a main strategies for fish to adapt to low oxygen environments (Shang et al., 2022). Our RNA-Seq data increased the depth and coverage of gene expression patterns for *A. nigrocauda* under acute hypoxia stress for different lengths of time. This is our first study on changes in the transcriptome in heart and brain tissues in response to hypoxia and the following re-oxygenation.

Heart is a vital organ involved in oxygen transport and distribution, and therefore is especially sensitive to changes in oxygen levels (Motyka et al., 2017). Previous transcriptomic studies about hypoxia stress in different fishes indicated genes enriched in cell proliferation (Marques et al., 2008), glycolysis (Xia et al., 2018) and innate immune process (Mu et al., 2020) were differentially expressed in heart tissue. The differential expression of genes across the different time points of hypoxia stress highlights the dynamic nature of the response to oxygen deprivation in heart tissue. In the initial phase of hypoxia (1.5 h vs. 0 h), a significant number of genes (466 DEGs) were differentially expressed. Several of these genes were associated with the Vascular smooth muscle contraction pathway, suggesting an early adaptive response occurred to maintain blood flow and oxygen supply. And this pattern is in accordance with mammal heart under hypoxia, which is accompanied by modifications of the extracellular matrix composition and variations in cardiac muscle metabolism (Fago and Jensen, 2015). The number of DEGs was highest (739) after 3 h of hypoxia stress, indicating a heightened cellular response with prolonged stress. The number of DEGs decreased slightly (528) after 4.5 h of hypoxia stress; however, there was a notable increase of genes in the enriched MAPK and VEGF signaling pathways. Both these pathways have crucial roles in cellular proliferation, differentiation, and survival under stress conditions (Jopling et al., 2012; Xia et al., 2018; Mu et al., 2020). The consistent upregulation of *hif1al* across all three hypoxia-stressed groups underscores its pivotal role in hypoxia adaptation. Hif1al is a well-known regulator of cellular responses to low oxygen levels (Mu et al., 2020; Yan et al., 2021). And this gene is also found continuously regulated during the process of hypoxia in brain tissue of juvenile yellow catfish (*Pelteobagrus fulvidraco*) (Wang et al., 2021). Above all, the gene expression pattern under hypoxia stress is similar to that in *Larimichthys crocea* although its duration (6h, 24h and 48 h) is much longer than that in this study (Mu et al., 2020).

The re-oxygenation groups showed intriguing patterns of changes in the transcriptome. The most significant transcriptomic change was in the 1.5 h vs. Re1.5 h comparison, with a staggering 2,210 DEGs. This finding suggests that the initial re-oxygenation after the shortest hypoxia exposure time triggered a robust cellular response, possibly to repair any damage and restore normalcy. Some of these DEGs were annotated with the GO terms response to abiotic stimulus and regulation of response to stress, further emphasizing the transcriptomic response to counteract the effects of hypoxia (Yan et al., 2021). Interestingly, the number of DEGs identified during re-oxygenation decreased at longer hypoxia exposure times. The 3 h vs. Re3h comparison had the least number of DEGs (131), suggesting a potential adaptive mechanism where prolonged hypoxia leads to a more controlled and less drastic response upon re-oxygenation (Wang et al., 2021). The increase of DEGs in the enriched Apoptosis and mTOR signaling pathways in the re-oxygenation phase is particularly noteworthy. Apoptosis, or programmed cell death, is a critical process that eliminates damaged cells, and its activation suggests a mechanism to remove cells that might have been irreparably harmed during hypoxia. The mTOR signaling pathway, is central to cellular growth and survival, and its activation may be indicative of the transcriptomic response to promote cell survival and counteract the effects of hypoxia (Zhang et al., 2022).

The brain, which is one of the most oxygen-sensitive organs in fish, responded differently to hypoxia compared with the response in the heart. Initial exposure to hypoxia (1.5 h vs. 0 h) resulted in a relatively low number of DEGs (45), which is significantly fewer than the 466 DEGs observed in the heart tissue under similar conditions. This finding suggests that the initial response of the brain to hypoxia may be more conservative or regulated differently than that of the heart (Xia et al., 2018). In the 3 h vs. 0 h comparison, the number of DEGs increased to 311, indicating a heightened response to prolonged hypoxic stress. Notably, the enriched PPAR signaling pathway has been associated with lipid metabolism and inflammation in other organisms (Wang et al., 2021). When compared with a similar study in silver carp brain tissue, we found immune system related pathways, such as natural killer cell-mediated cytotoxicity and antigen processing and presentation were not presented in our result here (Feng et al., 2022). This might be caused by behavior difference between the two species and the low hypoxia tolerance for silver carp (Feng et al., 2022).

After re-oxygenation, the transcriptomic response was more pronounced in the brain tissue, especially in the 3 h vs. Re3h comparison with 2,865 DEGs, compared with the response in the heart tissue. This finding may be indicative of the heightened sensitivity of the brain to oxygen fluctuations and its critical role in maintaining homeostasis (Shang et al., 2022). The enriched mTOR signaling and FoxO signaling pathways suggest there may be a complex interplay of growth, survival, and metabolic processes in the brain during re-oxygenation (Qi et al., 2020). Interestingly, although some DEGs were commonly upregulated or downregulated across the different time points in both tissues, there was minimal overlap among the DEGs in the heart and brain tissues. Only one gene, *angptl4*, was commonly downregulated in both tissues, emphasizing the tissue-specific responses to hypoxia and re-oxygenation. Angptl4 (angiopoietin-like 4) is a multifunctional protein that has been implicated in various physiological and pathological processes (Aryal et al., 2019). *Angptl4* has been shown to be regulated in response to low oxygen conditions. Specifically, Pichiule et al. (Pichiule et al., 2004) found that the expression of hypoxia-driven *Ang2*, which is closely related to *Angptl4*, may be independent of the HIF pathway, suggesting that other mechanisms may be involved in regulating *Angptl4* under hypoxic conditions.

In transcriptomic analysis, Weighted Gene Co-Expression Network Analysis (WGCNA) serves as a pivotal tool for studying the complexity of gene expression patterns, grouping genes into modules based on similar expression profiles across different conditions or treatments. This approach simplifies the analysis of extensive gene expression data and helps in identifying networks of co-expressed genes that share biological functions or pathways. Based on WGCNA result from heart tissue, we found the key hub genes in purple module were mainly involved in signaling transduction, such as insulin receptor signaling pathway (GO:0008286). Signal transduction system is the regulator for body’s tolerance to hypoxia, and its role is applied by modulating transcription and translation, which is a vital factor for adaption to hypoxia stress in aquatic animals (Zhang et al., 2016; Yang et al., 2018). The insulin receptor signaling pathway manipulates the absorb of glucose in cell, promote the storage of different energy molecular in the form of glycogen in tissues, and maintains the balance of lipid and carbohydrate utilization (Eckel and Reinauer, 1990; Teleman, 2009). In these multiple procedures, oxygen is a vital element to transfer different lipid or carbohydrate into ATP for the survival and functioning of cells. And this pattern was also found in another WGCNA on hypoxia stress in *Larimichthys crocea* (Zhang et al., 2021).

We also carried out a large-scale analysis of AS events in brain and heart tissues of *A. nigrocauda*. In the brain tissue, we found that 4,510 of 9,902 genes (45.54%) had only one type of AS; the other 5,392 genes had multiple AS events. In the heart tissue, we found that 4,459 of 9,817 genes (45.42%) had only one AS events This pattern suggests that more multiple AS events than single AS events occurred under environment pressure, which is consistent with findings in other fishes. For example, 103 differential AS genes were found in heart tissue of Nile tilapia under hypoxia stress (Xia et al., 2018), and 162 differential AS events were detected in liver tissue of spotted sea bass under high salinity conditions (Tian et al., 2020). These results indicate the involvement of AS events as a support to DEGs may help fishes cope with various environmental stresses (Vu et al., 2022).

## 5 Conclusion

Our comprehensive analysis of the transcriptomic responses of *A. nigrocauda* heart and brain tissues under hypoxia and subsequent re-oxygenation identified intricate molecular adaptations. The discovery of significant alterations in key signaling pathways, combined with the identification of numerous new transcripts and genes, underscores the complexity of the *A. nigrocauda* response to oxygen deprivation. Furthermore, the prominence of AS events highlights an additional layer of regulatory mechanisms that are used by fish under hypoxia stress. Together, our findings not only enhance the understanding of the molecular strategies used by *A. nigrocauda* to cope with hypoxic conditions, but also emphasize the potential of these insights to inform strategies for aquatic conservation and fisheries management.

## Data Availability

The datasets presented in this study can be found in online repositories. The names of the repository/repositories and accession number(s) can be found below: https://www.ncbi.nlm.nih.gov/bioproject/PRJNA1027844.
