# Supplementary figures and images for: Transcriptomic atlas for hypoxia and following re-oxygenation in Ancherythroculter nigrocauda heart and brain tissues: insights into gene expression, alternative splicing, and signaling pathways
Source: Front Genet. 2024 Apr 16;15:1365285. doi: 10.3389/fgene.2024.1365285 (PMC11058841; doi:10.3389/fgene.2024.1365285)

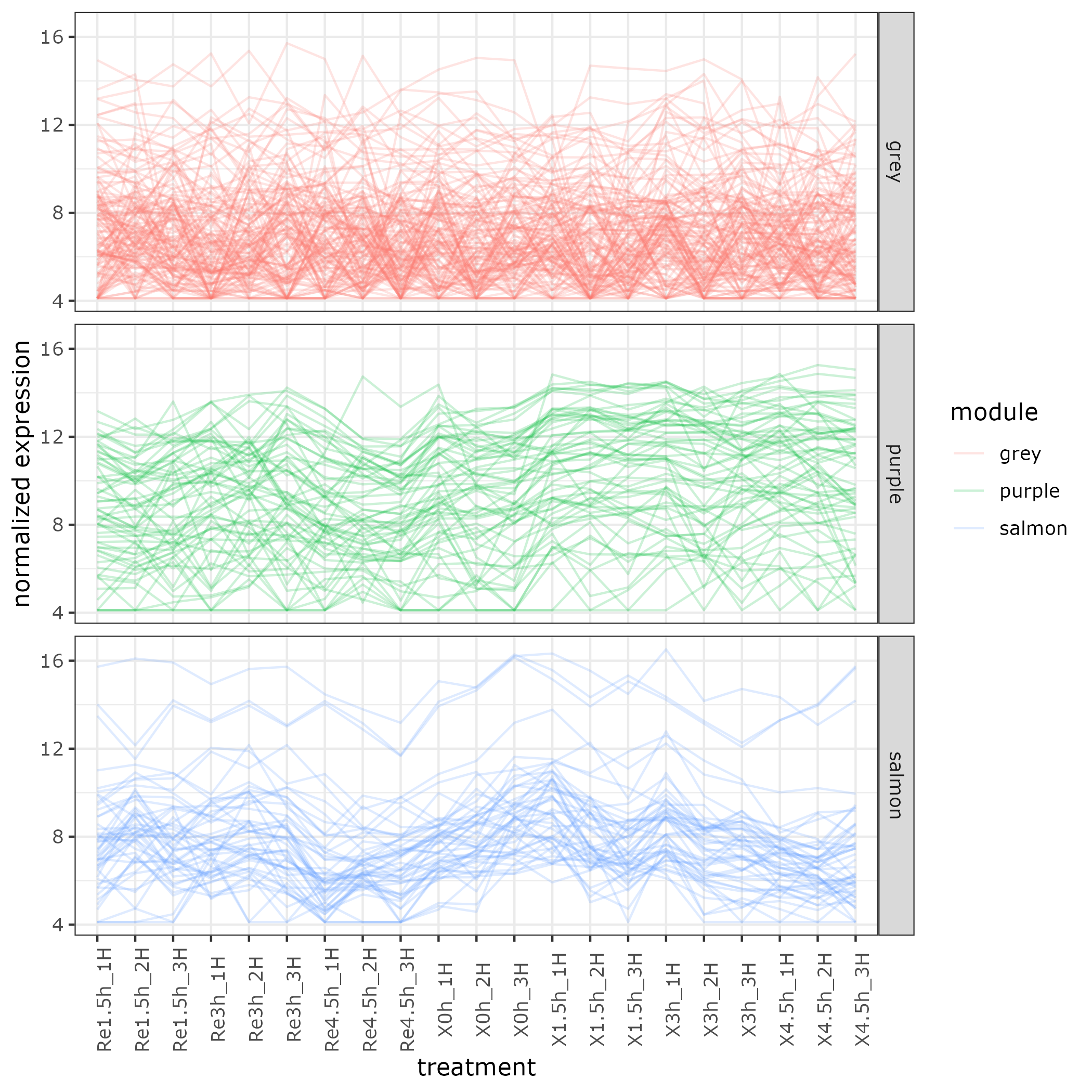

Supplement: Supplementary file 1 [file Image6.TIF]

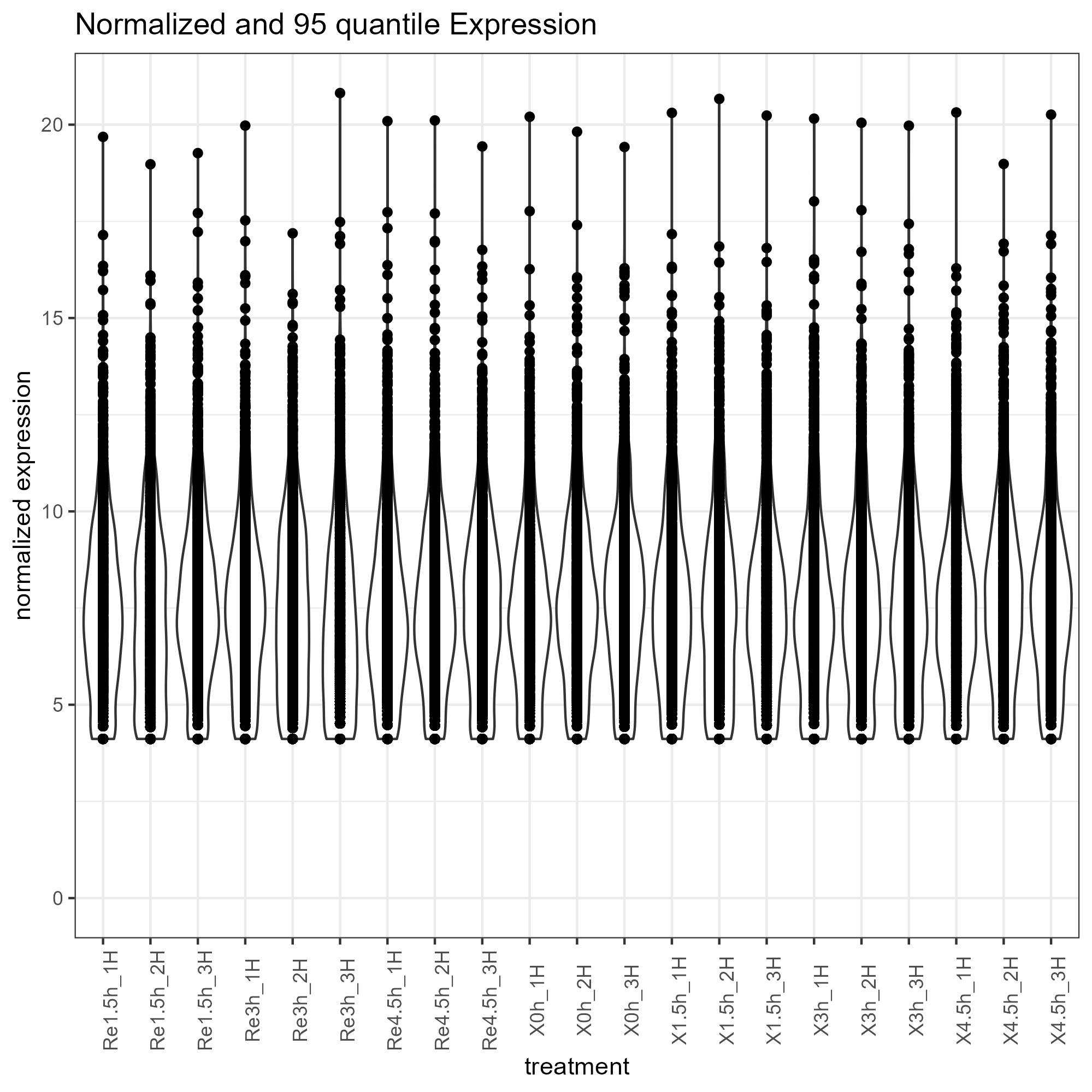

Supplement: Supplementary file 2 [file Image3.TIF]

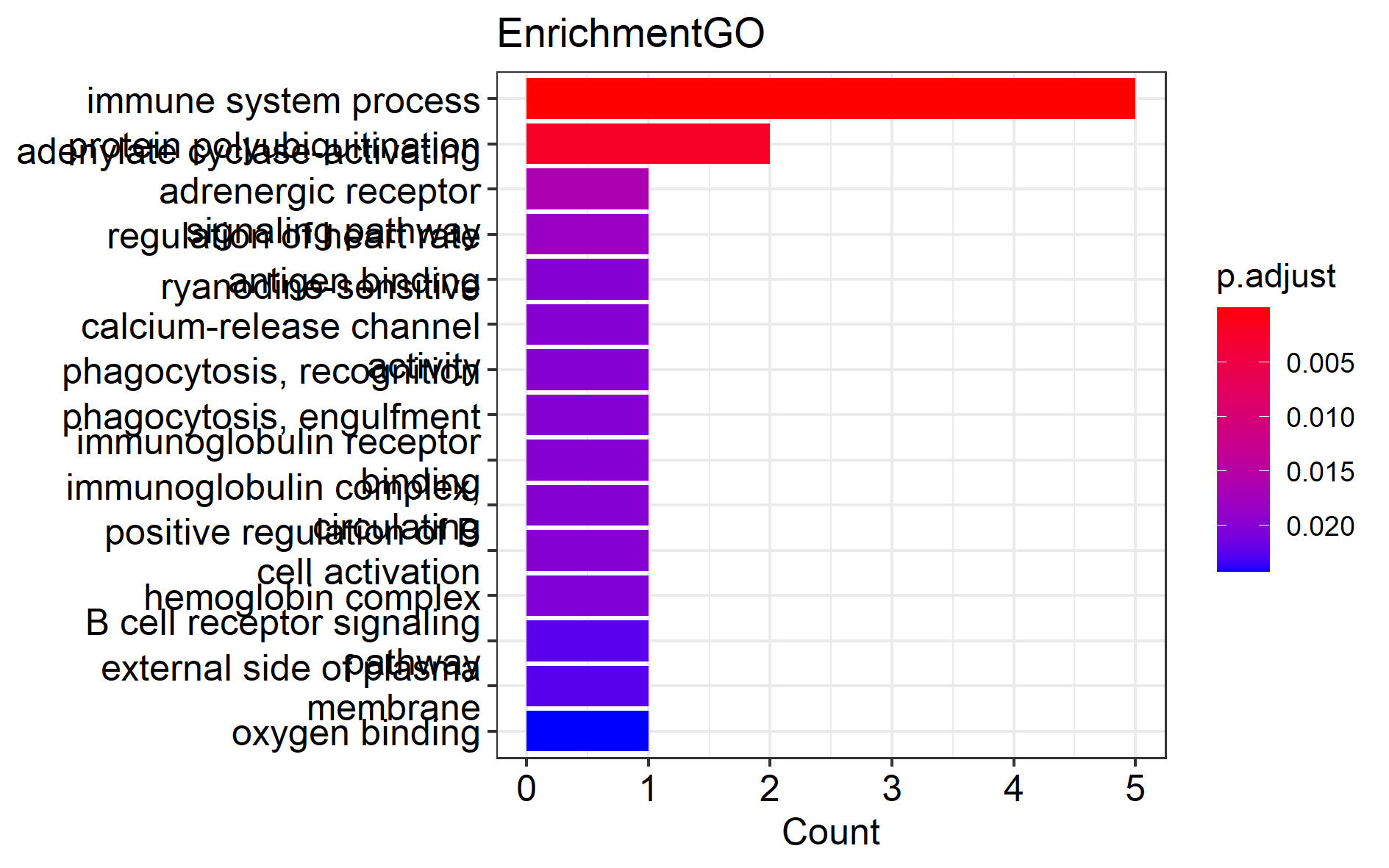

Supplement: Supplementary file 3 [file Image5.TIFF]

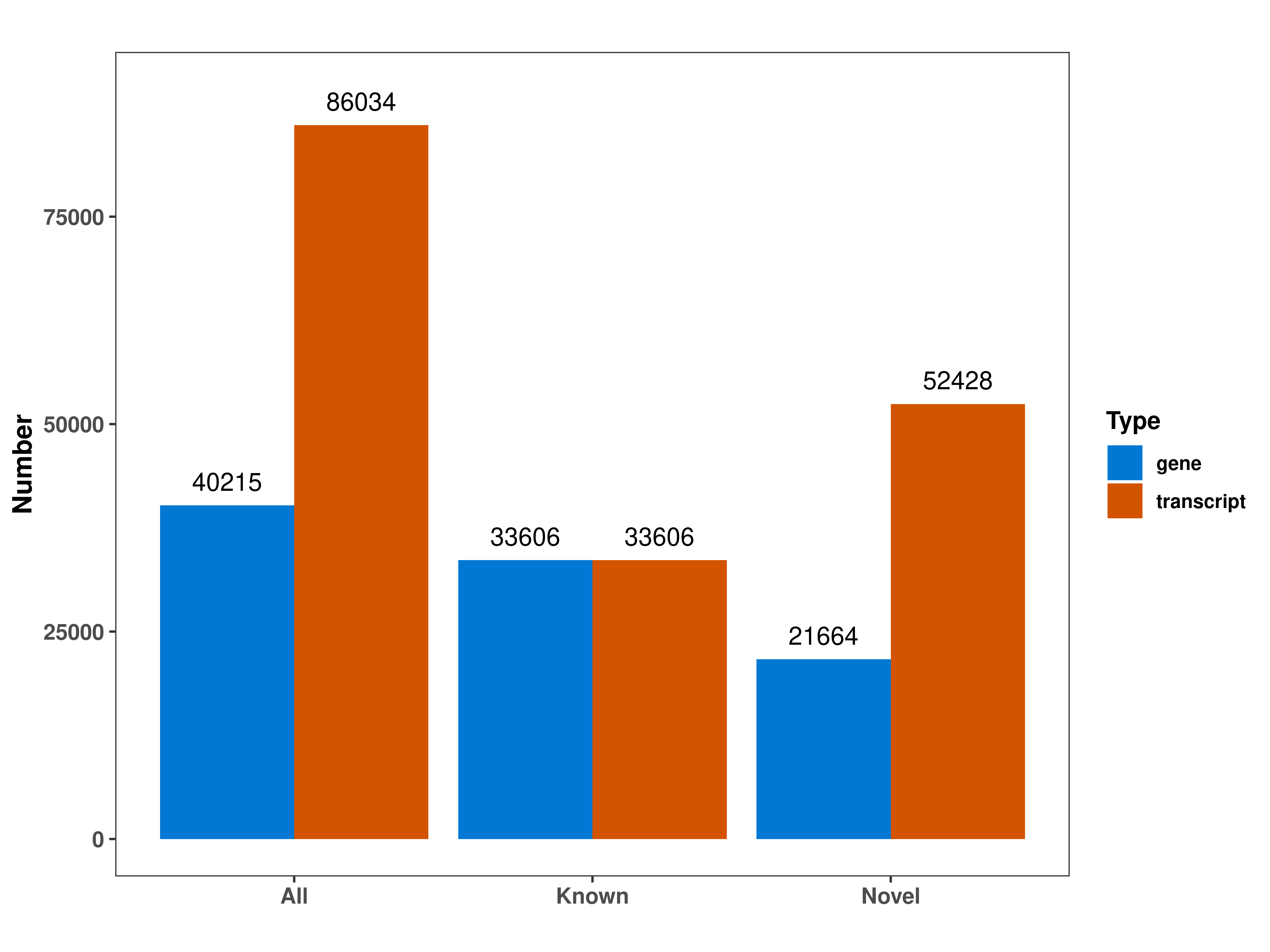

Supplement: Supplementary file 4 [file Image2.PNG]

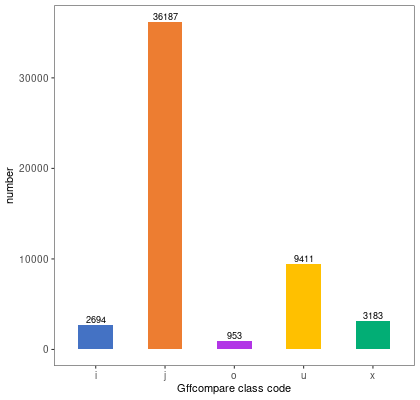

Supplement: Supplementary file 6 [file Image1.PNG]

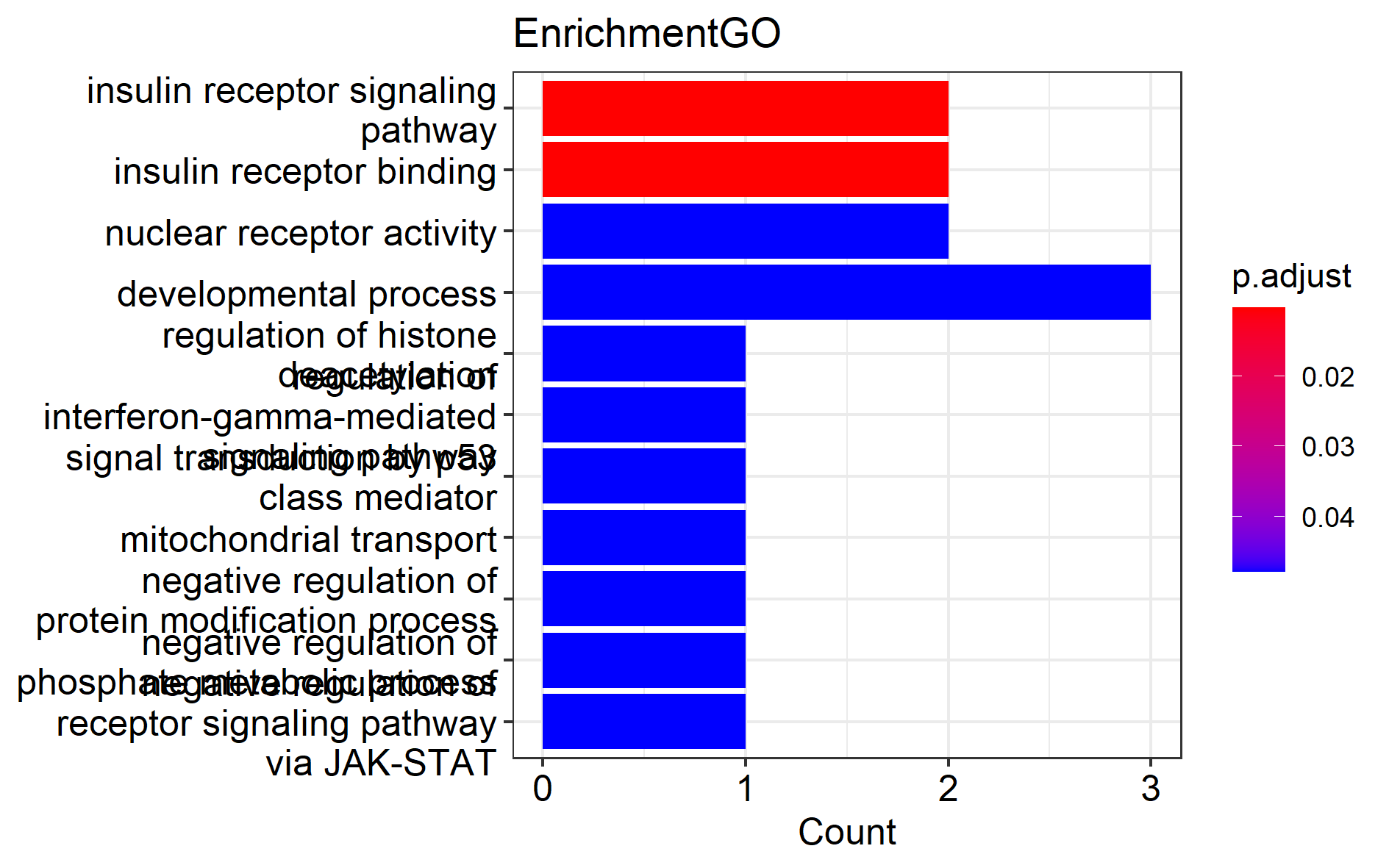

Supplement: Supplementary file 7 [file Image4.TIFF]
